# Oral diseases and systemic conditions: correlation analyses from the Colombian national health records between 2016 and 2023

**DOI:** 10.3389/froh.2024.1466427

**Published:** 2024-09-25

**Authors:** Margarita Usuga-Vacca, David Díaz-Báez, Edgar O. Beltrán, Andrea Cortes, Paula Katherine Vargas-Sanchez, Viviana Avila

**Affiliations:** ^1^ UNICA—Caries Research Unit, Research Department, Universidad El Bosque, Bogotá, Colombia; ^2^ UIBO—Unit of Basic Oral Investigation, School of Dentistry, Universidad El Bosque, Bogotá, Colombia; ^3^ Postgraduate Program of Periodontics and Oral Medicine, Universidad El Bosque, Bogotá, Colombia

**Keywords:** oral disease, dental caries, periodontitis, non-communicable diseases, public health, socioeconomic disparities

## Abstract

**Introduction:**

Prevalence of oral, metabolic, circulatory, and nutritional diseases has tended to increase globally. As these diseases have common contributing factors, such as unhealthy diets, evaluating their epidemiological trends and the relation between them is relevant. In Colombia, the Ministry of Health records the frequency of consultation for these diseases through the Integrated Social Protection Information System. It facilitates the evaluation of their epidemiological behavior at the population level.

**Aim:**

To analyze and correlate the burden of oral diseases (caries and periodontitis) with respect to nutritional, circulatory and metabolic pathologies, as well as their relationships with socioeconomic indices in Colombian regions from 2016 to 2023.

**Methods:**

This study analyzes retrospective data collected between 2016 and 2023 by the National Health Registry in Colombia (SISPRO) according to the ICD-10. Sociodemographic data and the number of disease consultations were extracted. The number of consultations for oral diseases was correlated with systemic pathologies, socioeconomic indices through the Spearman test. Associations were explored via multiple linear regressions. A Principal Component Analyses (PCA) was conducted to identify patterns between socioeconomic, oral and systemic disease variables, as well as to reduce the complexity of the analysis by creating clusters that capture the greatest possible variability in the data.

**Results:**

The average number of consultations per biennium was 24.550.435 being the highest number for 2018–2019. The highest percentage of consultations was related to extensive caries, followed by chronic gingivitis. Moreover, consultations for oral diseases were found to correlate with systemic pathologies. All socioeconomic indices were associated with caries and/or periodontal diseases. This study is the first in Colombia that uses national data on diagnoses assigned to medical and dental consultations. PCA revealed a clustering pattern of pathologies suggesting that they are highly associated one to each other.

**Conclusion:**

Correlations between consultations for oral and systemic diseases stratified by life cycle and socioeconomic indices highlight the importance of conducting epidemiological and public health characterizations and their associations. Identifying these factors is imperative in the prevention and management of both diseases.

## 1 Introduction

More than 3.5 billion people worldwide were affected by oral diseases in 2019. The World Health Organization (WHO) reported that almost half of the world's population suffers from untreated oral diseases (dental caries and periodontal diseases), representing the most prevalent condition among more than 300 systemic diseases and conditions that affect humanity (1). Because of those diseases, affection of quality of life, productivity and exacerbate socioeconomic inequalities (i.e., the greatest burden of disease has been found in disadvantaged and marginalized population groups) have been reported (2–4).

Dental caries is a non-communicable disease (NCD) that occurs in primary teeth and affects 514 million children worldwide. The corresponding figure for permanent teeth is 2 billion people globally. It is an important public health problem for populations and governments (1, 5). In addition to dental caries, periodontal disease also has a high prevalence globally in people over 15 years of age, reaching its peak at approximately age 55 and remaining high until adulthood and later in life. The prevalence across country income groups is similar, but the number of cases is highest in lower-middle-income countries (437 million) and lowest in low-income countries (80 million) (6, 7). Oral diseases share determinants and risk factors with other NCDs (8). Their presence is determined by the behaviors that people adopt (9, 10), leading to systemic diseases such as heart disease, stroke, and chronic lung diseases (1, 11–13).

The characterization of poverty and social factors related to general and oral health/diseases has been described using various methods, such as the Multidimensional Poverty Index (MPI), the Human Development Index (HDI) and the Gini index. For example, the MPI uses a range of indicators to calculate a summary poverty figure for a given population, for which a larger figure indicates a higher level of poverty (14). It uses health, education and standard of living indicators to determine the incidence and intensity of poverty or deprivation experienced in a population. The MPI is reported as a percentage ranging from the lowest deprivation score (less poor) to the highest deprivation score (poorest). On the other hand, the HDI corresponds to an average of the subnational values of three dimensions: education, health and standard of living (15). Finally, the Gini index is a measure of statistical dispersion intended to represent income inequality, wealth inequality, or consumption inequality within a nation or a population. A Gini index of 0 reflects perfect equality, whereas a higher Gini index reflects maximal inequality among values (16).

The Comprehensive Information System for Social Protection (SISPRO, for its initials in Spanish) consists of databases and information systems on the supply and demand of health services. SISPRO includes the reports of the information recorded using the International Classification of Diseases, Tenth Edition (ICD-10) (17), which are accessible with the permission of the Colombian Ministry of Health and Social Protection (SISPRO).

In Latin American countries such as Colombia, the factors mentioned above have not been explored in depth via SISPRO data. Consequently, based on the theoretical conceptual framework of the Commission on Social Determinants of Health, the aim of this work was to analyze and correlate the burden of oral diseases (caries and periodontitis) with respect to nutritional, circulatory and metabolic pathologies, as well as their relationships with socioeconomic indices in Colombian regions from 2016 to 2023.

## 2 Materials and methods

An analysis of retrospective real-life data was conducted. Data were collected from the National Health Registry in Colombia, Comprehensive Social Protection Information System (SISPRO) (www.sispro.gov.co). Data extraction was conducted between January 2016 and December 2023. In this study, temporal and ecological trend approaches were used to analyze the associations between the number of consultations for oral diseases and systemic pathologies and the socioeconomic indicators of the different Colombian regions. The current geographical division of the Colombian territory is based on 32 departments (provinces) and Bogotá (Capital District), which act as administrative units. All the regions were grouped into six regions (Caribbean, Pacific, Coffee Axis, South Central, Los Llanos and Central East). In this study, diseases were classified based on the International ICD-10. The number of consultations for oral diseases (initial caries, K020; extensive caries, K021, K023; root caries, K022; other dental caries, K028 and K029; acute gingivitis, K050; chronic gingivitis, K051; acute periodontitis, K052; chronic periodontitis, K053; and other periodontal diseases, K055) was recorded. General diseases were also recorded: nutritional—malnutrition, E40 to E46; other nutritional deficiencies, E50 to E64; obesity and other types of hypernutrition, E65 to E68; metabolic diseases—diabetes mellitus, E10 to E14; and circulatory system diseases, I00 to I99.

Other explanatory variables included age, which was categorized from the data available in the SISPRO, into life cycles considering the WHO classification [Infancy (0–04 years), Childhood (05–09 years), Adolescence (10–19 years), Young Adulthood (20–29 years), Adulthood (30–59 years) and Later life (≥60 years)] (World Health Organization & Ministerio de Salud y Protección Social). Sex (female/male) and residence region were used as keys to nest socioeconomic indices (human development, poverty and income inequality). Socioeconomic indices were assessed as follows: databases of multidimensional poverty indicators from the MPI, the Integrated Household Survey of the National Administrative Department of Statistics (DANE), and the Gini index. The DANE in Colombia is responsible for planning, surveying, processing, analyzing and disseminating official statistics. HDI data were obtained from the Global Data Lab (GDL) system. The IMP data represent the economic situation for the periods studied (2018–2019, 2020–2021 and 2022–2023), whereas the Gini and HDI data represent income inequality and human development, respectively, for the periods 2016–2017, 2018–2019 and 2020–2021; only the available data by department were considered. The included socioeconomic indicators were classified as follows: MPI, lowest deprivation score (<33%); deprivation score (>33%–50%); and highest deprivation score (>50%–100%). The HDI included low (<0.550), medium (0.550–0.699), high (0.700–0.799) and very high (>0.800) categories, and the Gini index for inequality assessment was low (<0.30), moderate (>0.30–<0.50) or high (>0.50). All the variables mentioned above were considered to assess their impact on oral health.

### 2.1 Analytical strategy

The sum of the total number of patients consulting for each evaluation period (biennium) was calculated by life course, sex, and oral diseases as dependent variables, and explanatory systemic conditions were included. Similarly, the total number of people consulted was estimated in relation to the MPI, HDI and Gini index categories. Descriptive analysis was performed to calculate the proportions of consultations between each category included.

An exploratory analysis was performed to verify the consistency of the data, and the collinearity between the independent variables was assessed. Subsequently, three different analyses were performed. First, a Spearman correlation analysis was conducted to verify the relationship between the explanatory variables and the dependent variables (bivariate analysis) and visualize the effect of the temporal trend. This analysis verified correlations between a given factor and the outcome and if this association was maintained over time. The results were staged as low (values ranging: 0–0.39), moderate (values ranging: 0.4–0.69) and high (above: ≥0.7) correlations (12).

A second approach aimed to assess the effect of each explanatory variable, considering the confounding effects of the analysis variables among themselves. Therefore, these variables were included in five multiple linear regression analysis models to verify the association between them (a model for each type of oral disease). Prior to the regression analysis, the following variable control was applied: The Kolmogorov‒Smirnov was used to assess normality; heteroskedasticity in the distribution of these variables was estimated with the Kendall-Stuart and Goldfeld-Quandt tests; data were corrected for heteroskedasticity and non-normality of the distribution of the variables using the Box‒Cox power transformations. Explanatory variables that presented a *p* value ≤ 0.20 in the correlation assessment with each outcome were included in the model fit (18). This assessment is part of the exploratory phase of the study and allowed the researchers to explore potential associations more broadly before refining the model.

A third approach consisted of a principal component analysis (PCA), which was based on weighting and aggregating the common data characteristics. This approach can be presented as a “dimension reduction tool” and “an analysis of identifying the data pattern” and is widely used to condense a large set of variables into a small number of variables while maintaining the proportional weight of the variables (19). The main objective of the application of PCA was to identify underlying patterns between socioeconomic, oral and systemic disease variables, as well as to reduce the complexity of the analysis by creating clusters that capture the greatest possible variability in the data. PCA identified the grouping of the number of consultations for pathologies according to their correlations and saturations in two main dimensions, highlighting the connections between oral diseases and systemic pathologies and socioeconomic factors. All analyses were processed and performed using Stata 14 (StataCorp®, Microsoft Power BI, 2020) and IBM SPSS Statistics V.22 (IBM Corp®).

## 3 Results

The average number of attended consultations extracted from the SISPRO database and included for the final analyses of this study (2016‒2023) was 24.550.444. Table 1 shows the biennial distribution details according to the life course, sex, oral diseases related to caries and periodontitis and systemic diseases (including metabolic and nutritional conditions). The percentage of consultations was highest for women in later life. Across all time intervals, the highest percentages of oral disease consultations were related to extensive caries (>49%), followed by chronic gingivitis (>19%) and acute gingivitis (>12%). With respect to systemic diseases, the percentages were consistent across each interval, with consultations for hypertensive diseases being the most common, followed by consultations for diabetes, obesity and other types of malnutrition.

**Table 1:** Characteristics of the biennial distribution of the number of medical consultations in terms of life course, sex, and incidence of oral and systemic diseases.

	Years							
	2016–2017		2018–2019		2020–2021		2022–2023	
	*n* = 20,268,279	(%)	*n* = 30,111,389	(%)	*n* = 24,338,447	(%)	*n* = 23,483,623	(%)
Life courses								
Infancy	57	(0.00)	12,336	(0.04)	185,635	(0.76)	484,478	(2.06)
Childhood	315,322	(1.56)	1,175,370	(3.90)	1,087,044	(4.47)	1,043,860	(4.45)
Adolescence	1,947,926	(9.61)	2,961,673	(9.849	1,924,869	(7.91)	1,828,739	(7.79)
Young Adulthood	2,312,065	(11.41)	3,304,254	(10.97)	1,901,793	(7.81)	1,839,467	(7.83)
Adulthood	6,955,190	(34.32)	10,611,204	(35.24)	8,384,676	(34.45)	8,423,146	(35.87)
Later life	8,737,719	(43.11)	12,046,552	(40.01)	10,854,430	(44.60)	9,863,933	(42.00)
Sex								
Female	12,261,220	(60.49)	18,002,077	(59.78)	14,648,877	(60.19)	14,265,773	(60.75)
Male	8,007,059	(39.51)	12,109,312	(40.22)	9,689,570	(39.81)	9,217,850	(39.25)
Oral diseases								
Initial caries	355,160	(3.67)	616,062	(4.23)	300,686	(3.35)	247,812	(2.7)
Extensive caries	4,842,481	(50.11)	7,228,379	(49.61)	4,802,029	(53.44)	5,037,706	(55.7)
Root caries	82,170	(0.85)	138,225	(0.95)	97,522	(1.09)	89,928	(1.0)
Other dental caries	481,669	(4.98)	661,908	(4.54)	409,379	(4.56)	474,290	(5.2)
Acute gingivitis	1,475,901	(15.27)	2,037,040	(13.98)	1,234,110	(13.73)	1,132,766	(12.5)
Chronic gingivitis	2,172,059	(22.47)	3,481,180	(23.89)	1,798,674	(20.02)	1,741,131	(19.3)
Acute periodontitis	51,181	(0.53)	84,010	(0.58)	81,781	(0.91)	62,627	(0.7)
Chronic periodontitis	161,781	(1.67)	272,826	(1.87)	218,222	(2.43)	209,441	(2.3)
Other periodontal diseases	42,164	(0.44)	51,195	(0.35)	43,109	(0.48)	44,181	(0.5)
Systemic diseases								
Malnutrition	252,363	(2.4)	362,131	(2.3)	296,738	(1.9)	332,798	(2.3)
Obesity and other types of hypernutrition	1,062,564	(10.0)	1,742,287	(11.2)	1,500,317	(9.8)	1,353,298	(9.4)
Other nutritional deficiencies	68,150	(0.6)	145,952	(0.9)	164,193	(1.1)	191,998	(1.3)
Diabetes mellitus	1,889,658	(17.8)	2,801,132	(18.0)	3,043,693	(19.8)	2,708,777	(18.8)
Hypertensive diseases	5,027,744	(47.4)	6,909,349	(44.5)	7,294,217	(47.5)	6,816,020	(47.2)
Acute rheumatic fever	3,975	(0.0)	5,655	(0.0)	4,757	(0.0)	1,780	(0.0)
Arteries, arterioles and capillar diseases	124,634	(1.2)	206,124	(1.3)	176,926	(1.2)	177,057	(1.2)
Cardiopulmonary and pulmonary circulation diseases	35,750	(0.3)	52,937	(0.3)	54,849	(0.4)	50,229	(0.3)
Cerebrovascular diseases	213,596	(2.0)	339,002	(2.2)	345,704	(2.3)	335,557	(2.3)
Chronic rheumatic and heart diseases diseases	20,465	(0.2)	33,929	(0.2)	30,911	(0.2)	31,431	(0.2)
Diseases of veins and lymphatic vessels and nodes, not elsewhere classified	1,001,498	(9.4)	1,410,125	(9.1)	1,025,063	(6.7)	1,082,840	(7.5)
Ischemic heart diseases	352,491	(3.3)	589,055	(3.8)	567,066	(3.7)	550,639	(3.8)
Other circulatory system disorders (non-specified)	30,993	(0.3)	59,969	(0.4)	53,054	(0.3)	41,863	(0.3)
Other forms of heart disease	519,832	(4.9)	882,917	(5.7)	795,447	(5.2)	769,454	(5.3)

The total number of consultations evaluated, revealed that the Caribbean region experienced an increase of 31.1% between 2016‒2017 and 2018‒2019, stabilizing at approximately 21%‒22% of the total consultations in all periods. The Central East region, the most prominent region in terms of the absolute number of consultations (32.6%‒37.9%), showed sustained growth, reaching 37.9% in 2022‒2023. The most notable increase was 29.0% from 2016‒2017 to 2018‒2019. The Coffee Axis had an initial increase of 33.5% from 2016‒2017 to 2018‒2019, followed by a decrease, reflecting a declining percentage distribution from 22.3% to 17.7%. Los Llanos showed constant growth, reaching 3.0% in 2022‒2023. The Pacific region experienced fluctuations, increasing by 29.9% between 2016‒2017 and 2018‒2019 but ending at 13.2% in 2022‒2023. The South-Central region showed notable growth until 2018‒2019 at 30.4%, followed by a decrease, representing between 6.0% and 7.6% of the total consultations (Supplementary Table 1).

As the main findings in the analysis of the distribution of oral diseases among the regions of Colombia from 2016‒2023, significant variations in the prevalence of consultations were observed. Overall, the Central East region presented extensive caries, initial caries, root caries, acute gingivitis, acute periodontitis, chronic gingivitis, and chronic periodontitis. For extensive caries, the proportion of cases in the Central East region remained high, fluctuating around 34%‒35%, whereas the Caribbean region showed a gradual decrease from a maximum of 32.8% in 2016‒2017 to 28.4% in 2022‒2023. The prevalence of initial caries in the Central East region steadily increased, reaching 41.3% in the 2022‒2023 period, in contrast to the Caribbean region, where this rate decreased from 37.8% initially to approximately 22.8% at the end of the period. The prevalence of root caries also increased in the Central East region, remaining at approximately 35.3% at the end of the study period, whereas other regions, such as the Caribbean and Coffee Axis, presented lower proportions (between 17% and 22%) (Supplementary Table 2).

Acute gingivitis was particularly predominant in the Central East region, reaching 58.1% in 2022‒2023, whereas in the Caribbean region, it decreased from 25.4% in 2016‒2017 to 10.4% in 2022‒2023. The Coffee Axis showed a notable proportion of acute periodontitis, which initially was 24.1% in 2016‒2017. The Pacific region showed consistency in the prevalence of extensive caries and initial caries, maintaining proportions of approximately 14%–19%. Although these conditions were less prominent in Los Llanos region, although with lower proportions, an increment in root caries and chronic gingivitis were observed, reaching approximately 2.7% and 1.5%, respectively, from 2022 to 2023. The South-Central region showed a stable trend for most diseases, with slight decreases in initial caries and chronic periodontitis (Supplementary Table 2).

The Caribbean and Los Llanos regions face higher levels of poverty, especially in rural areas, whereas the Central East and Coffee Axis show better human development indicators. In terms of the sociodemographic indicators among the regions of Colombia from 2018 to 2023, the average total MPI was highest in the Caribbean region (31.39 in 2018‒2019, 28.79 in 2020‒2021, and 21.52 in 2022‒2023). This index was the lowest in the Central East and Coffee Axis regions (13.57 and 13.53 in 2018‒2019, respectively, which decreased to 9.65 and 9.74 in 2022‒2023, respectively). The urban MPI followed a similar trend, with the Caribbean and Los Llanos regions showing the highest averages. The rural MPI was significantly greater in all regions, especially in the Caribbean (46.98 in 2018‒2019) and Los Llanos region (39.65 in 2018‒2019), although these values tended to decrease. The HDI was highest in the Central East and Coffee Axis regions, with averages of 0.77‒0.78 in 2018‒2019, whereas other regions, such as the Pacific and Los Llanos regions, had slightly lower averages, approximately 0.73‒0.74. The Gini coefficients showed the highest inequality in the Pacific region (0.51 in 2020‒2021) (Supplementary Table 3).

The number of patients treated for oral diseases strongly and significantly correlated with the number of patients included in the analyses (correlation value >0.7; *p* value <0.05). As presented in Table 2, the strongest correlations were found between initial/root caries, chronic gingivitis, obesity and other types of hypernutrition (correlation value >0.9). Moderate correlations were found between this type of caries and acute rheumatic fever, ischemic heart diseases and malnutrition (correlation values between 0.4 and <0.7). Strong correlations were also found between initial caries but with lower values. Moderate correlations were found between initial caries and chronic periodontitis, acute rheumatic fever, cardiopulmonary and pulmonary circulation diseases, diabetes mellitus, cerebrovascular diseases, hypertensive diseases, ischemic heart diseases, malnutrition, and other and unspecified disorders of the circulatory system; the correlation was weakest for ischemic heart diseases. Root caries was strongly correlated with almost all the included diseases, with the highest value associated with extensive caries. Moderate correlations were found with ischemic heart diseases, malnutrition and other nutritional deficiencies.

**Table 2:** Correlation of the number of patients treated for oral diseases with the other diseases included in the analysis.

	Extensive caries	Initial caries	Root caries	Chronic gingivitis	Chronic periodontitis
Extensive caries	–				
Initial caries	.931**	–			
Root caries	.902**	.865**	–		
Chronic gingivitis	.947**	.892**	.868**	–	
Chronic periodontitis	.787**	.668**	.822**	.793**	–
Acute rheumatic fever	.690**	.668**	.709**	.651**	.638**
Arteries, arterioles and capillar diseases	.816**	.703**	.824**	.814**	.924**
Cardiopulmonary and pulmonary circulation diseases	.774**	.682**	.750**	.795**	.811**
Cerebrovascular diseases	.735**	.637**	.778**	.739**	.911**
Chronic rheumatic and heart diseases	.822**	.766**	.852**	.804**	.835**
Diseases of veins, lymphatic vessels, and lymph nodes, not classified elsewhere	.846**	.754**	.852**	.842**	.940**
Hypertensive diseases	.719**	.620**	.752**	.728**	.905**
Ischemic heart diseases	.606**	.472**	.671**	.614**	.872**
Diabetes mellitus	.747**	.642**	.782**	.740**	.929**
Malnutrition	.694**	.694**	.580**	.672**	.382**
Obesity and other types of hypernutrition	.931**	.847**	.873**	.912**	.861**
Other circulatory system disorders (non-specified)	.762**	.649**	.804**	.760**	.909**
Other forms of heart disease	.829**	.757**	.846**	.827**	.904**
Other nutritional deficiencies	.797**	.758**	.696**	.753**	.563**

Analysis performed using spearman correlation.

Number is Rho Spearman value and **p < 0.05.

Chronic gingivitis strongly correlated with most conditions (*p* < 0.05) except for acute rheumatic fever, ischemic heart diseases and malnutrition, for which correlations were moderate. Chronic periodontitis was strongly correlated with diabetes mellitus; diseases of arteries, arterioles, and capillaries; and diseases of veins, lymphatic vessels, and lymph nodes not classified elsewhere; and hypertensive diseases (*p* < 0.05). In contrast, chronic gingivitis disease was moderately correlated with acute rheumatic fever and other nutritional deficiencies (*p* < 0.05). In addition, malnutrition and chronic gingivitis exhibited the only mild correlation Table 2.

Table 3 shows information related to the distribution of individuals who consulted for nutritional, circulatory and metabolic pathologies related to oral diseases in Colombia from 2016 to 2023. The data are categorized by biennial periods, MPI, urban and rural classifications, HDI, and the Gini index. Missing data were not available in the source of information used.

**Table 3:** Number of consultations for nutritional, circulatory and metabolic pathologies related to oral diseases in Colombia from 2016 to 2023, categorized by biennial periods (2016–2017, 2018–2019, 2020–2021, and 2022–2023).

Index	Options	Consultation number from 2016 to 2023 by biennial period			
		2016–2017	2018–2019	2020–2021	2022–2023
MPI	Lowest deprivation	No data	27,234,282	22,837,170	22,987,311
	Deprivation		2,440,170	533,046	464,932
	Highest deprivation		436,937	316,564	23,188
MPI—urban	Lowest deprivation	No data	30,078,973	24,310,173	23,456,202
	Deprivation		32,416	28,274	27,421
	Highest deprivation		0	0	0
MPI—rural	Lowest deprivation	No data	15,075,214	18,362,308	19,789,424
	Deprivation		14,016,773	4,313,662	2,793,541
	Highest deprivation		1,019,402	428,033	363,446
HDI	Low	0	0	0	No data
	Medium	95,587	163,797	157,168	
	High	16,550,354	24,936,483	19,841,297	
Gini	Low inequality	3,622,338	5,011,109	0	No data
	Moderate inequality	9,805,159	14,034,718	7,317,362	
	High inequality	6,840,782	13,427,116	12,675,046	

**Figure 1:**
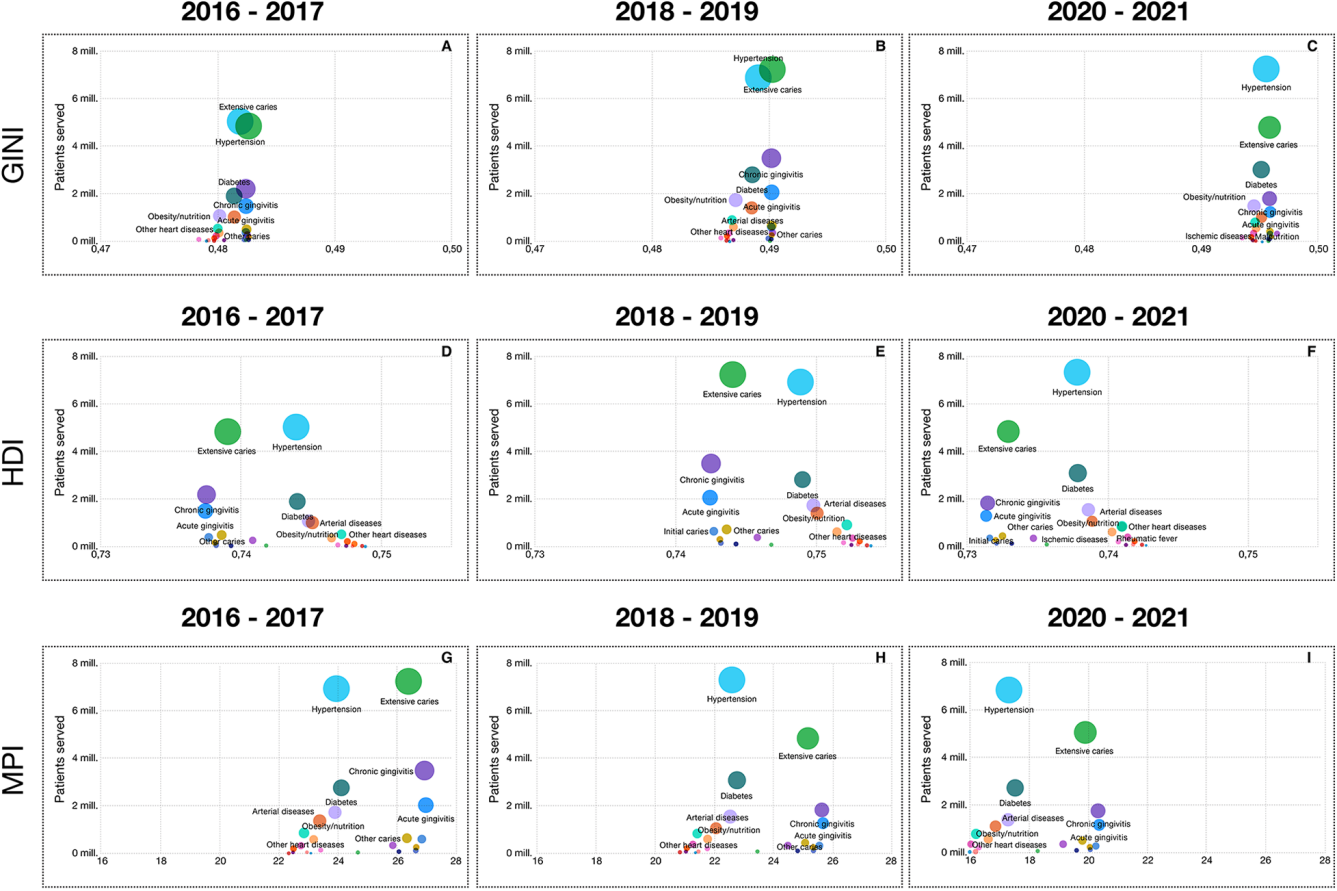
Relationships between socioeconomic indices and the number of attended consultations for oral and systemic diseases (2016–2023). The figure shows the relationship between different socioeconomic indices (GINI, HDI, and MPI) and the number of patients who attended consultations for various oral and metabolic conditions in three periods: 2016–2017, 2018–2019, and 2020–2021. The panels are organized in a three-row by three-column matrix, with each row representing a socioeconomic index and each column representing a time period: **(A)** GINI 2016–2017, **(B)** GINI 2018–2019, **(C)** GINI 2020–2021, **(D)** HDI 2016–2017, **(E)** HDI 2018–2019, **(F)** HDI 2020–2021, **(G)** MPI 2016–2017, **(H)** MPI 2018–2019, **(I)** MPI 2020–2021. In each graph, the *Y*-axis represents the number of attended consultations (in millions), while the *X*-axis shows the value of the corresponding index. The evaluated oral and metabolic conditions are represented by colored bubbles, where size is proportional to the number of patients who attended consultations for that specific condition.

According to the MPI, individuals residing in urban areas experiencing the lowest deprivation constituted the largest proportion of consultations. With respect to the HDI, the category with the highest number of inquiries was the lowest deprivation segment, which presented more consultations during the second biennium included in this study. Based on available Gini index data, the most frequent consultations were classified as highly inequality except for during the 2016‒2017 period, where most consultations were in regions with moderate inequalities. Figure 1 shows the relationships between socioeconomic indices (Gini, HDI and MPI) and the prevalence of various oral and systemic diseases in Colombia over four biennial periods (2016‒2023). The diseases with the highest number of consultations for diseases, such as extensive caries and arterial hypertension, consistently dominated in all periods and throughout the three indices. These two diseases increased in number among patients from 2016‒2017 to 2018‒2019, especially in contexts of high inequality and low HDI. This trend continued in 2020‒2021, with a notable concentration of pathologies in areas with high MPIs.

Table 4 shows the correlations between socioeconomic indices (Gini, HDI and MPI) and the pathologies for which consultations were recorded. Inequality and initial caries showed a weak but significant correlation. The same type of correlation was identified between level of development of the regions (HDI) and various pathologies: nutritional, metabolic and circulatory diseases correlated with dental diseases, such as coronal caries (initial and extensive), root caries, chronic gingivitis and periodontitis. Regarding the level of individual poverty (MPI), inverse and weak correlations were found for most of the pathologies, except for obesity and other types of hypernutrition, cardiopulmonary and pulmonary circulation diseases, which exhibited moderate correlations.

**Table 4:** Correlations of the number of attended consultations for oral and systemic diseases with the socioeconomic indices Gini index, HDI and MPI.

	Gini	HDI	MPI
Initial caries	.120**	.293**	−.269**
Extensive caries	.028	.346**	−.386**
Root caries	.052	.235**	−.265**
Chronic gingivitis	.016	.392**	−.425**
Chronic periodontitis	.045	.215**	−.237**
Acute rheumatic fever	−.017	.252**	−.099**
Arteries, arterioles and capillar diseases	−.008	.343**	−.349**
Cardiopulmonary and pulmonary circulation diseases	.057	.385**	−.469**
Cerebrovascular diseases	.005	.260**	−.264**
Chronic rheumatic and heart diseases diseases	−.024	.314**	−.258**
Diabetes mellitus	−.008	.252**	−.269**
Diseases of veins, lymphatic vessels, and lymph nodes, not classified elsewhere	−.004	.306**	−.343**
Hypertensive diseases	.003	.248**	−.280**
Ischemic heart diseases	.006	.186**	−.198**
Malnutrition	.023	.355**	−.392**
Obesity and other types of hypernutrition	−.020	.395**	−.446**
Other circulatory system disorders (non-specified)	−.009	.262**	−.224**
Other forms of heart disease	−.023	.322**	−.334**
Other nutritional deficiencies	.061	.343**	−.363**

Analysis performed using spearman correlation.

Number is Rho Spearman value and ** *p* < 0.05.

**Figure 2:**
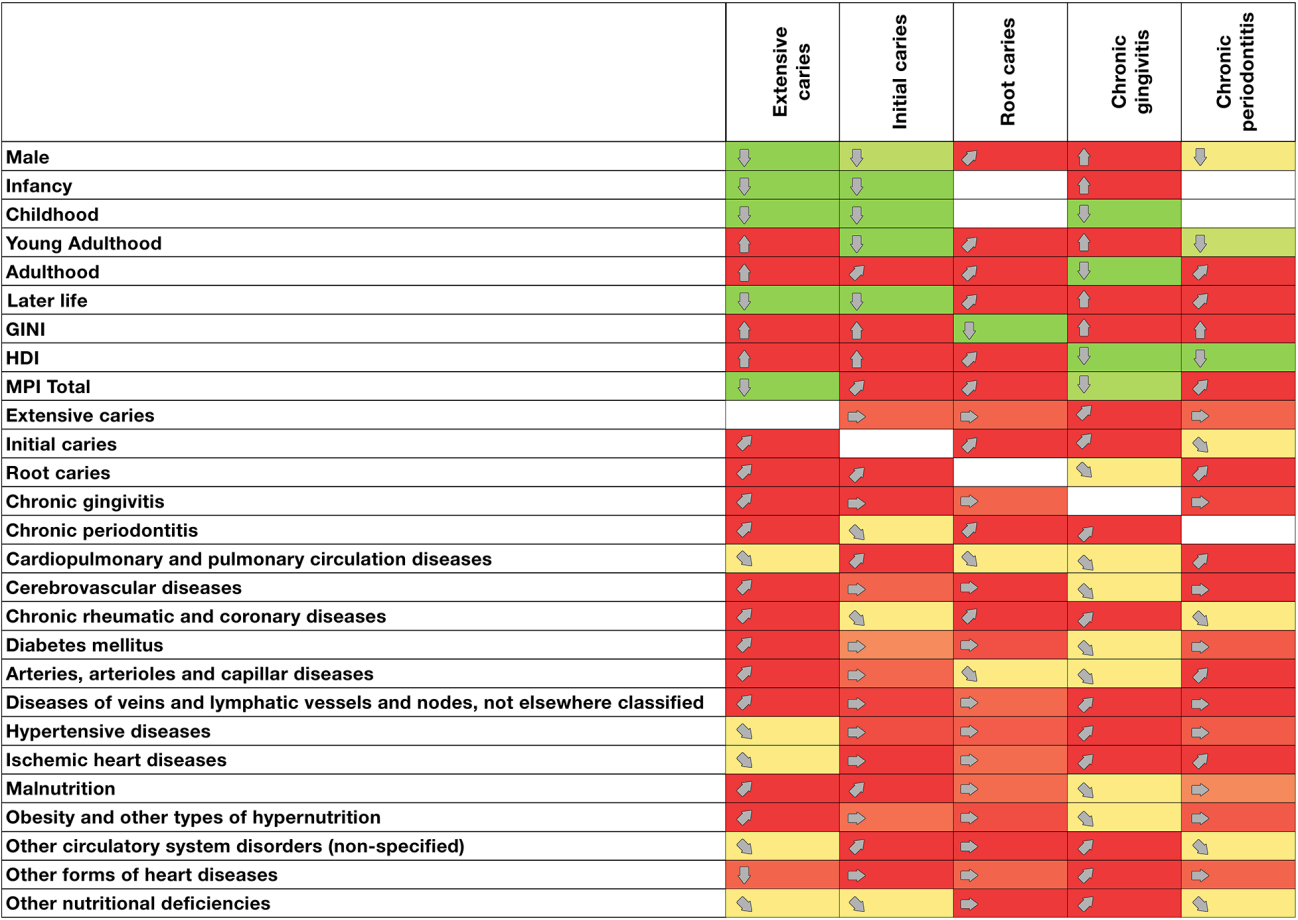
Association matrix between oral and metabolic pathologies and sociodemographic and health factors in Colombia (2016–2023). Colors: light green: Significant decrease in the number of health condition consultations. Light red: significant increase in the number of health condition consultations. Yellow: no significant change in the prevalence of health condition consultations. White: no data available or not applicable. Direction: (↓): significant and sustained decrease in the number of consultations; (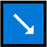): downward trend in the number of consultations; (→): no significant change or relationship in the number of consultations; (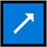): upward trend in the number of consultations; (↑): significant and sustained increase in the number of consultations.

The direction and strength of the associations are shown in Figure 2. For example, the probability of extensive caries consultation was greater in young adulthood and adulthood and correlated with economic inequalities and the level of human development. On the other hand, chronic rheumatic and coronary diseases tended to increase the number of caries and chronic gingivitis consultations.

Multiple linear regressions revealed significant associations between the evaluated factors and the number of patients with initial, extensive and root caries; chronic gingivitis; and chronic periodontitis (Supplementary Table 4). For chronic gingivitis, the positive coefficient (2,195.05, *p* = 0.003) indicated that men have a greater prevalence of consultations for this condition than women. With respect to the life course, several associations with oral health conditions were observed. Infancy and childhood were significantly associated with several conditions. For example, childhood presented a strong negative association with consultation for extensive caries (Coef. = −7,913.00, *p* < 0.001). Similarly, infancy showed significant negative associations with consultation for extensive (Coef. = −7,731.42, *p* < 0.001) and initial (Coef. = −697.34, *p* < 0.001) caries. In contrast, young people showed significant negative associations with both initial caries (Coef. = −349.03, *p* = 0.008) and chronic periodontitis (Coef. = −133.12, *p* = 0.047). The results also revealed relevant associations for the elderly population; in the case of initial caries, the *β* coefficient was negative and significant (Coef. = −632.21, *p* = 0.001). For chronic gingivitis, a positive coefficient (2,195.05, *p* = 0.003) was observed for sex.

Socioeconomic indicators revealed significant associations with the prevalence of oral disease consultations. The Gini coefficient, an indicator of economic inequality, showed a strong positive association with extensive caries (Coef. = 37,496.92, *p* = 0.007) and a negative association with root caries (Coef. = −1,335.81, *p* = 0.003). With respect to the socioeconomic indices, the HDI showed a significant positive relationship with initial caries (Coef. = 6,267.37, *p* = 0.007. The MPI was significantly associated with almost all conditions, including a strong negative association with extensive caries and chronic gingivitis (Coef. = −226.50, *p* < 0.001; −188.85 *p* = 0.003).

Systemic pathologies are also significantly associated with the incidence of oral diseases. For example, acute rheumatic fever was significantly associated with extensive caries (Coef. = 56.26, *p* < 0.001), which could indicate a relationship between these conditions. Cardiopulmonary and pulmonary circulation diseases are significantly associated with initial caries (Coef. = 0.84, *p* = 0.035) and other oral conditions. Malnutrition was significantly associated with extensive caries (Coef. = 3.17, *p* < 0.001). Similarly, obesity and other types of hypernutrition are significantly associated with extensive caries (Coef. = 1.16, *p* < 0.001).

The results of the five models (extensive caries, initial caries, root caries, chronic gingivitis and chronic periodontitis) indicated that the models were highly significant, with *p* values < 0.0001 in all cases. The *R*^2^ values obtained are high (0.9548 for severe caries, 0.8460 for initial caries, 0.9284 for root caries, 0.8561 for chronic gingivitis and 0.9731 for chronic periodontitis).

**Figure 3:**
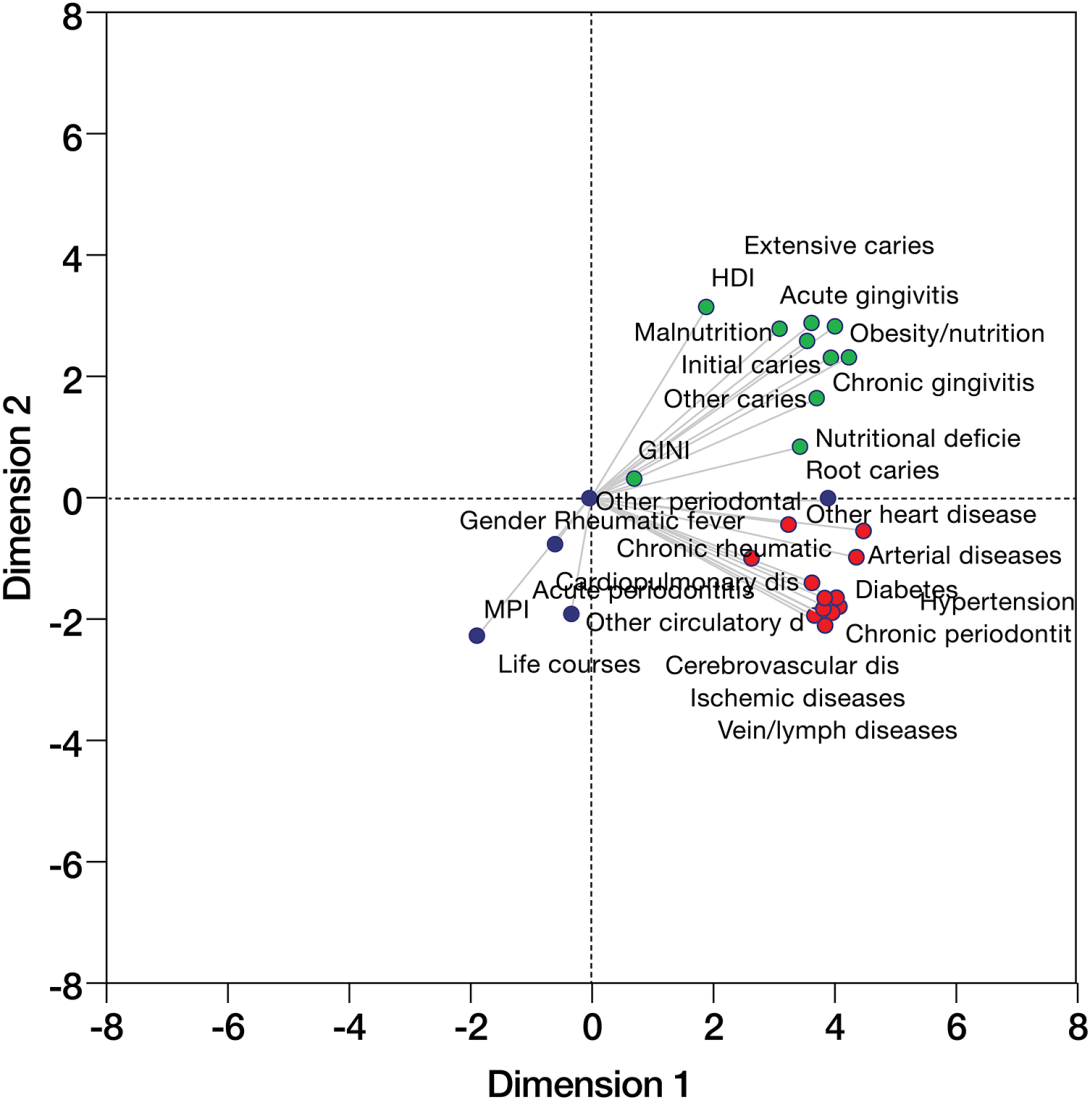
Principal component analysis (PCA) of different oral and systemic diseases, as well as sociodemographic factors of patients treated in the Colombian national health system from 2016 to 2023 is displayed in Figure 3. The upper right quadrant (green) includes oral and metabolic diseases with associated saturations. The lower left quadrant (blue) contains sociodemographic and MPI factors. The lower right quadrant (red) includes chronic diseases (including periodontitis) and cardiovascular diseases.

The PCA biplot results are presented in Figure 3, where the first two dimensions explained a significant portion of the variability of the data (>80%); the results of the model in Dimensions 1 and 2 presented Cronbach's alpha values of 0.97 and 0.831, respectively, indicating high robustness of the extracted dimensions. In addition to analyses based on the HDI and Gini index, the PCA revealed how pathologies tended to cluster according to their saturations in both dimensions. For example, attended consultations for oral diseases, such as extensive caries and chronic gingivitis, are strongly associated with each other and with metabolic pathologies such as obesity, nutritional disorders, and diabetes. On the other hand, negative saturation in Dimension 1 and negative saturation in Dimension 2 included variables such as sex, life cycles and MPI, suggesting an inverse effect on the prevalence of care for the diseases observed in positive Dimension 1. In the lower right quadrant, circulatory and metabolic diseases were clustered with chronic periodontitis. Negative saturations in both dimensions suggest that these chronic pathologies are highly associated with each other, possibly exacerbated by unfavorable socioeconomic and demographic conditions.

## 4 Discussion

This study focused on the analysis of correlations between the burden of oral diseases (caries and periodontal diseases) and nutritional, circulatory and metabolic pathologies during the 2016‒2023 period. The main findings revealed epidemiological trends and relationships. Specifically, the number/frequency of consultations for oral diseases correlated with nutritional, circulatory and metabolic pathologies, and the consulting population was classified by life cycle and socioeconomic indices. The number of consultations was highest in 2018‒2019 and lowest in 2016‒2017. With respect to oral diseases, the highest percentage of consultations were related to extensive caries, followed by chronic gingivitis. Correlations between consultations for oral diseases and systemic pathologies were identified. All socioeconomic indices were associated with caries and/or periodontal diseases.

This is the first study in Colombia that uses national data (SISPRO) on diagnoses assigned to medical and dental consultations (linking caries and periodontal diseases) throughout the registration period and seeks possible explanations for these associations based on age and living conditions (e.g., inequality, poverty, education level, and health). This type of exploration can improve the understanding of disease behavior in terms of the number of consultations for oral and systemic pathologies and can help to elucidate how oral and systemic health are connected to socioeconomic factors. In addition, public health decision-makers can support the development of initiatives that favor the control of common risk factors for these health conditions. This exploration could be useful as a reference for similar analyses in other Latin American countries.

The available information from the SISPRO system is limited to the number of consultations in the health system, which are classified by oral or systemic diseases (ICD-10); therefore, the results of this study should not be confused with reports on the prevalence of pathologies. Additionally, the assignment of morbidity diagnostic codes may not be standardized due to differences in diagnostic criteria among health professionals, lack of training or the academic level of individuals that filled out the records (undergraduates, doctors, medical students or specialists), which may hinder the ability to identify more precise relationships between pathologies. Although bivariate and multivariate analyses, such as PCA were used, these methods have limitations, such as multicollinearity and heteroscedasticity, which could affect the validity of the regression models (20).

In this study, the correlations between consultations for oral diseases and different oral pathologies were robust. These results were consistent across the different analyses, including the association analysis. This strong association may be because both dental and periodontal diseases can be diagnosed at the time of the consultation, especially during the first consultation or control consultations. In Colombia, these consultations are conducted based on Resolution 003280, 2018 (Individual Health Care) according to the life cycle and are conducted annually starting at an age of 6 months and decrease in frequency to biennial consultations starting in teenage life (21).

A biological explanation of this correlation could be the lack of adequate control of the biofilm, which is a common factor for both conditions (22–25). In Resolution 003280, 2018, control was achieved through biannual prophylaxis and biofilm removal, starting in the first year of life in infancy and continued throughout childhood. This periodicity decreases to once a year in adolescence and once every 2 years starting in adulthood with scaling when needed since adolescence. Specifically for dental caries, these correlations could also be explained by the topical application of fluoride in varnish, which is conducted starting the first year of life and continued every six months until adolescence (21). Additionally, the diagnosis of extensive caries refers to untreated caries, which is described as the most common health condition, at least in the permanent dentition, according to the WHO report (26).

Compared with consultations for oral pathologies, the consultation for chronic non-communicable systemic diseases, such as those of the circulatory system and diabetes, in were high in most cases. Exceptions included ischemic heart diseases and acute rheumatic fever, which showed moderate correlations. Previous studies have described a relationship between these systemic and oral pathologies (21, 23, 25–30); some of these associations occur through common risk factors, such as sugar consumption (31). Importantly, the periodicity of measures to control chronic non-communicable pathologies ranges from 3 to 12 months, depending on the state of control of the pathology, which is greater when the indicators are at appropriate levels for age (32–34).

Among nutritional and metabolic pathologies, only the number of consultations for malnutrition showed moderate correlations with caries and gingivitis and weak correlations with consultations for periodontitis. Previous reports have described how nutritional factors, such as high sugar consumption, are associated with other pathologies, both systemic and oral, in addition to obesity (35–37). This correlation highlights the importance of interventions for oral diseases, which could have an impact on the number of patients with systemic diseases that assist and are treated.

The biennial distribution of consultations in the health system from 2016 to 2023 revealed a greater frequency of consultation among the older adult population and female individuals in all biennial periods. In both groups, consultations may be more frequent because these populations represent almost half (45.5%) and more than half (60%) of patients with chronic diseases, respectively, and these individuals regularly attend health services to control their illness (38).

With respect to consultations for systemic non-communicable pathologies, obesity predominates among nutritional pathologies. This finding has been reported as a global epidemic by the WHO, especially for middle-income countries (such as Colombia) (39). This condition could increase the risk for cardiovascular diseases, such as hypertension, and metabolic diseases, such as diabetes mellitus (40). The latter condition was responsible for the highest number of consultations in this study.

In this study, the socioeconomic characteristics of the regions where the consultations took place correlated with the prevalence of systemic and oral pathologies in each biennial period. This pattern suggests a strong correlation between socioeconomic inequalities and the prevalence of consultations for these health conditions, especially in areas with greater economic inequality and multidimensional poverty.

Consultation for hypertension and extensive caries predominated in all biennial periods, especially in the contexts of low human development (HDI), high rates of multidimensional poverty (MPI) and high inequality (Gini). The levels of human development were directly correlated with all groups of pathologies included in this study (nutritional, circulatory, metabolic and oral), whereas multidimensional poverty was inversely correlated with these indices. These results reflect the clear relationship between morbidity and socioeconomic factors in the population from which the data were obtained, as previously described (41). According to the Division for Heart Disease and Stroke Prevention of the Centers for Disease Control and Prevention (CDC), socioeconomic factors can affect health status and can even interact with/or confound relationships between other variables and health (42). For example, low socioeconomic status is associated with a greater risk of developing cardiovascular diseases (43). Additionally, evidence supports a relationship between oral diseases and socioeconomic factors, with the poorest and marginalized groups being the most affected (44).

The socioeconomic factors and number of total consultations were highly correlated with the lowest indices of deprivation, the highest human development index, and moderate to high levels of inequality. This finding is consistent with that of Carr-Hill et al., who affirmed that socioeconomic factors “can act as powerful predictors of consultation patterns” (45). In line with the previous approach, socioeconomic factors are recognized as barriers to accessing health services/care (46).

With respect to the PCA associations, three clusters of associations were found. The first cluster included caries, gingivitis, nutritional pathologies, the Gini index and the HDI. The second cluster included circulatory diseases, diabetes and chronic periodontitis. The third cluster included demographic factors, the MPI, and other periodontal and non-defined circulatory diseases. These associations have been previously described between cardiovascular, circulatory and periodontitis diseases and other systemic diseases, including diabetes and respiratory diseases (23, 30, 47). Although these analyses included explanatory and outcome variables, establishing a direct association is not possible, as in some cases, a strictly defined diagnosis is found in SISPRO.

## 5 Conclusion

The burden of oral diseases (caries and periodontitis) was correlated with nutritional, circulatory and metabolic pathologies, as well as with socioeconomic indices in Colombian regions from 2016 to 2023.

## Data Availability

The raw data supporting the conclusions of this article are available in the Integrated Social Protection Information System SISPRO databases.
