# Supplementary material for: Oral diseases and systemic conditions: correlation analyses from the Colombian national health records between 2016 and 2023
Source: Front Oral Health. 2024 Sep 25;5:1466427. doi: 10.3389/froh.2024.1466427 (PMC11461449; doi:10.3389/froh.2024.1466427)
Supplement: Supplementary file 1 [file Datasheet1.pdf]

Supplementary Material

Supplementary table 1. Number of medical consultations by region and biennial periods (2016–2017, 2018–2019, 2020–2021, and 2022–2023).

	2016-2017	2018-2019	2020-2021	2022-2023
Caribbean	4285437	6637103	5188728	5201979
Central East	6665186	9818455	8455275	8896589
Coffee Region	4524760	6827572	4841771	4165244
Los Llanos	468723	814498	687139	712941
Pacific	2967737	4101834	3561152	3096022
South-Center	1356436	1911927	1604382	1410848

Supplementary table 2. Number of medical consultations for each oral disease by region and biennial periods (2016–2017, 2018–2019, 2020–2021, and 2022–2023).

	Caribbean	Central East	Coffee Region	Los Llanos	Pacific	South-Center
**2016-2017**						
Acute gingivitis	374479	501267	153144	46970	158187	241854
Acute periodontitis	9233	19357	12353	801	5921	3516
Chronic gingivitis	393474	700980	733033	32580	213390	98602
Chronic periodontitis	14824	69204	48353	2221	18211	8968
Other dental caries	178106	89008	55889	13002	56564	89100
Extensive caries	26931	28616	6386	3066	13514	3657
Initial caries	134149	78376	47565	8656	67572	18842
Root caries	1014874	1805424	850929	142045	722399	306810
**2018-2019**						
Acute gingivitis	507189	644379	307519	79001	203326	295626
Acute periodontitis	11596	37010	21115	1818	6876	5595
Chronic gingivitis	662445	1044397	1215058	67856	330866	160558
Chronic periodontitis	26702	122040	75261	4409	29337	15077
Other dental caries	322475	142328	76296	6680	73407	40722
Extensive caries	51807	49942	13476	5101	12711	5188
Initial caries	213029	175392	79595	21264	87739	39043
Root caries	1669851	2598671	1219887	270405	978424	491141
**2020-2021**						
Acute gingivitis	323196	379354	157944	47246	140176	186194
Acute periodontitis	8904	45610	12799	1465	7508	5495
Chronic gingivitis	253805	795974	453897	27121	182777	85100
Chronic periodontitis	19858	111274	47367	3366	23886	12471

	Caribbean	Central East	Coffee Region	Los Llanos	Pacific	South-Center
Other dental caries	151560	143093	43318	4344	45639	21425
Extensive caries	18294	49754	12221	2308	8419	6526
Initial caries	88497	99742	34993	11371	43997	22086
Root caries	1068374	1853121	683892	181546	663425	351671
**2022 - 2023**						
Acute gingivitis	273529	388493	143728	49303	120782	156931
Acute periodontitis	6517	36394	10228	865	5473	3150
Chronic gingivitis	271800	788406	416393	24626	168026	71880
Chronic periodontitis	21680	115239	40249	2815	19426	10032
Other dental caries	110040	232071	33066	33902	44613	20598
Extensive caries	16963	48578	9980	3492	7220	3695
Initial caries	70262	86024	31376	12029	31003	17118
Root caries	1147080	2081429	683874	195006	588976	341341

Supplementary table 3. Multidimensional Poverty Index, Human Development Index, and Inequality (GINI) in Colombian regions and biennial periods (2016–2017, 2018–2019, 2020–2021, and 2022–2023).

	Caribbean	Central East	Coffee Region	Los Llanos	Pacific	South-Center
**MPI Total**						
**2018-2019**	31.39	13.57	13.53	30.22	24.82	22.35
**2020-2021**	28.79	12.84	13.05	29.64	22.16	22.26
**2022-2023**	21.52	9.65	9.74	25.44	17.26	15.31
**Urban MPI**						
**2018-2019**	23.64	9.88	9.53	21.28	16.17	15.34
**2020-2021**	21.32	9.58	9.68	21.56	13.70	15.19
**2022-2023**	14.96	7.38	7.02	18.19	11.29	10.60
**Rural MPI**						
**2018-2019**	46.98	25.82	27.97	39.65	33.48	33.98
**2020-2021**	43.28	24.42	26.05	39.59	31.44	33.88
**2022-2023**	33.88	18.18	20.94	32.72	23.99	23.28
**HDI**						
**2016-2017**	0.74	0.77	0.77	0.73	0.73	0.73
**2018-2019**	0.74	0.78	0.77	0.74	0.74	0.73
**2020-2021**	0.73	0.76	0.76	0.73	0.73	0.72
**GINI**						
**2018-2019**	0.47	0.47	0.48	0.47	0.51	0.49
**2020-2021**	0.48	0.48	0.47	0.49	0.52	0.49
**2022-2023**	0.49	0.51	0.48	0.48	0.51	0.49

Supplementary Table 4. Multiple linear regression revealed significant associations between the evaluated factors and the number of patients with extensive caries, initial caries, root caries, chronic gingivitis or chronic periodontitis.

		**Extensive caries**		**Initial caries**		**Root caries**		**Chronic gingivitis**		**Chronic periodontitis**	
		Parameters	Valor p	Parameters	Valor p	Parameters	Valor p	Parameters	Valor p	Parameters	Valor p
	Coef.	-7731.42		-697.34				2624.81			
Infancy	Std. Err.	1364.33	0.000	157.96	0.000			1467.49	0.074		
	t	-5.67		-4.41				1.79			
	Coef.	-7913.00		-303.61				-914.96			
Childhood	Std. Err.	1259.80	0.000	148.98	0.042			1367.71	0.504		
	t	-6.28		-2.04				-0.67			
Young Adulthood	Coef.	754.37		-349.03		64.49		1896.20		-133.12	
	Std. Err.	1148.11	0.511	130.55	0.008	36.36	0.077	1199.57	0.115	66.71	0.047
	t	0.66		-2.67		1.77		1.58		-2.00	
	Coef.	926.51		96.49		8.34		-1303.71		71.38	
Adulthood	Std. Err.	1323.40	0.484	151.47	0.524	42.04	0.843	1384.95	0.347	77.13	0.355
	t	0.70		0.64		0.20		-0.94		0.93	
	Coef.	-1875.24		-632.21		57.85		933.02		133.39	
Later life	Std. Err.	1641.79	0.254	186.06	0.001	52.14	0.268	1720.48	0.588	95.66	0.164
	t	-1.14		-3.40		1.11		0.54		1.39	
	Coef.	-306.90		-158.08		8.06		2195.05		-21.51	
Sex (Male)	Std. Err.	715.56	0.668	81.61	0.053	22.72	0.723	743.01	0.003	41.72	0.606
	t	-0.43		-1.94		0.35		2.95		-0.52	
	Coef.	2.17		0.00		0.15		4.25		-0.21	
Initial caries	Std. Err.	0.37	0.000	0.00	-	0.01	0.000	0.36	0.000	0.02	0.000
	t	5.83		0.00		14.99		11.92		-10.56	
	Coef.	0.00		0.03		0.01		0.25		0.01	
Extensive caries	Std. Err.	0.00	-	0.00	0.000	0.00	0.000	0.04	0.000	0.00	0.020
	t	0.00		5.83		5.12		5.61		2.34	
	Coef.	6.91		1.98		0.00		-14.48		0.87	
Root caries	Std. Err.	1.35	0.000	0.13	0.000	0.00	-	1.30	0.000	0.07	0.000
	t	5.12		14.99		0.00		-11.12		12.28	
	Coef.	0.23		0.05		-0.01		0.00		0.02	
Chronic gingivitis	Std. Err.	0.04	0.000	0.00	0.000	0.00	0.000	0.00		0.00	0.000
	t	5.61		11.92		-11.12		0.00		8.43	
	Coef.	1.76		-0.83		0.26		6.24		0.00	
Chronic periodontitis	Std. Err.	0.75	0.020	0.08	0.000	0.02	0.000	0.74	0.000	0.00	-
	t	2.34		-10.56		12.28		8.43		0.00	
Acute rheumatic fever	Coef.	56.26	0.000	-2.72	0.148	0.51	0.322	4.84	0.780	-4.85	0.000

		Extensive caries		Initial caries		Root caries		Chronic gingivitis		Chronic periodontitis	
		Parameters	Valor p	Parameters	Valor p	Parameters	Valor p	Parameters	Valor p	Parameters	Valor p
	Std. Err.	16.01		1.88		0.52		17.32		0.91	
	t	3.51		-1.45		0.99		0.28		-5.30	
Arteries, arterioles and capillar diseases	Coef.	1.71		-0.02		-0.20		-1.12		0.27	
	Std. Err.	1.44	0.234	0.16	0.911	0.04	0.000	1.50	0.457	0.08	0.001
	t	1.19		-0.11		-4.43		-0.74		3.26	
Cardiopulmonary and pulmonary circulation diseases	Coef.	-7.27		0.84		-0.20		-23.81		2.77	
	Std. Err.	3.46	0.036	0.40	0.035	0.11	0.074	3.48	0.000	0.16	0.000
	t	-2.10		2.12		-1.79		-6.84		17.17	
Cerebrovascular diseases	Coef.	1.78		-0.01		0.07		-2.65		0.06	
	Std. Err.	0.80	0.026	0.09	0.903	0.03	0.009	0.83	0.002	0.05	0.186
	t	2.23		-0.12		2.62		-3.19		1.32	
Chronic rheumatic heart diseases	Coef.	14.98		-1.44		0.46		6.36		-1.05	
	Std. Err.	5.50	0.007	0.63	0.023	0.17	0.009	5.79	0.272	0.32	0.001
	t	2.73		-2.29		2.64		1.10		-3.28	
Diabetes mellitus	Coef.	0.50		-0.06		0.01		-0.43		0.00	
	Std. Err.	0.17	0.003	0.02	0.001	0.01	0.030	0.18	0.015	0.01	0.864
	t	2.99		-3.27		2.18		-2.44		-0.17	
Diseases of veins, lymphatic vessels, and lymph nodes, not classified elsewhere	Coef.	0.31		0.03		-0.02		0.81		0.08	
	Std. Err.	0.19	0.103	0.02	0.170	0.01	0.001	0.20	0.000	0.01	0.000
	t	1.63		1.37		-3.38		4.14		8.15	
Hypertensive diseases	Coef.	-0.39		0.02		0.00		0.17		-0.02	
	Std. Err.	0.07	0.000	0.01	0.056	0.00	0.152	0.08	0.039	0.00	0.000
	t	-5.16		1.91		-1.44		2.07		-4.91	
Ischemic heart diseases	Coef.	-0.89		0.07		-0.02		0.37		0.11	
	Std. Err.	0.29	0.003	0.03	0.031	0.01	0.059	0.31	0.225	0.02	0.000
	t	-3.04		2.16		-1.89		1.22		6.79	
Malnutrition	Coef.	3.17		0.17		-0.02		-0.46		-0.06	
	Std. Err.	0.43	0.000	0.05	0.001	0.01	0.125	0.48	0.338	0.03	0.021
	t	7.33		3.30		-1.54		-0.96		-2.31	
Obesity and other types of hypernutrition	Coef.	1.16		-0.03		0.01		-0.21		0.00	
	Std. Err.	0.11	0.000	0.01	0.027	0.00	0.000	0.13	0.095	0.01	0.788
	t	10.68		-2.22		3.90		-1.67		0.27	
Other circulatory system disorders (non-specified)	Coef.	-1.44		0.36		0.05		4.34		-0.78	
	Std. Err.	1.78	0.419	0.20	0.074	0.06	0.345	1.85	0.020	0.10	0.000
	t	-0.81		1.79		0.94		2.34		-7.93	
Other forms of heart disease	Coef.	0.01		0.07		0.02		0.66		0.02	
	Std. Err.	0.26	0.955	0.03	0.019	0.01	0.071	0.28	0.017	0.02	0.299
	t	0.06		2.36		1.81		2.39		1.04	
Other nutritional deficiencies	Coef.	-1.44		-0.48		0.06		5.40		-0.18	
	Std. Err.	0.90	0.108	0.10	0.000	0.03	0.025	0.91	0.000	0.05	0.001
	t	-1.61		-4.73		2.24		5.94		-3.44	

		Extensive caries		Initial caries		Root caries		Chronic gingivitis		Chronic periodontitis	
		Parameters	Valor p	Parameters	Valor p	Parameters	Valor p	Parameters	Valor p	Parameters	Valor p
	Coef.	-226.50		25.28		4.56		-188.85		6.37	
MPI Total	Std. Err.	59.62	0.000	6.83	0.000	1.91	0.017	62.74	0.003	3.51	0.071
	t	-3.80		3.70		2.39		-3.01		1.81	
	Coef.	30297.08		6267.37		79.33		-27542.82		-1141.34	
HDI	Std. Err.	20151.52	0.133	2294.72	0.007	641.31	0.902	21108.13	0.193	1176.40	0.332
	t	1.50		2.73		0.12		-1.30		-0.97	
	Coef.	37496.92		2055.86		-1335.81		24364.93		301.67	
Gini index	Std. Err.	13965.58	0.007	1606.81	0.201	442.69	0.003	14683.22	0.098	819.79	0.713
	t	2.68		1.28		-3.02		1.66		0.37	

Analysis developed through multiple linear regression analysis. A model for each outcome of oral disease.
